# The research progress of LACC1

**DOI:** 10.3389/fimmu.2025.1698702

**Published:** 2025-11-25

**Authors:** Feng Li, Xinyi Cui, Qingli Yang, Yu Nie, Jinchun Wang, Shibo Sun

**Affiliations:** 1 Department of Pulmonary and Critical Care Medicine, First Affiliated Hospital, Kunming Medical University, Kunming, China; 2 Clinical Medicine, Kunming Medical University, Kunming, China

**Keywords:** LACC1, inflammation, immune, signaling pathway, diseases

## Abstract

Laccase Domain-Containing 1(LACC1)is highly expressed in myeloid macrophages, and plays a crucial role in coordinating innate and adaptive immune responses by integrating lipid, polyamine, and purine metabolism pathways. This study comprehensively discusses the molecular structure, enzymatic functions, and signaling pathways associated with LACC1. Notably, the M-CSF/AKT/mTOR/LACC1 pathway primarily regulates cellular autophagy. Additionally, the PPARα/RXR/PPRE/LACC1, miR-211-5p/KDM2B/LACC1, LACC1/AMPK/NLRP3, LACC1/NF-kB, LACC1/MAPK, LACC1/ROS/NOD2/PIPK2, LACC1/JAK-STAT, and LACC1/CCL5 signaling pathways are significantly involved in modulating inflammatory responses. Furthermore, the article provides a comprehensive summary of the pathogenic mechanisms and recent advancements in research concerning LACC1 in immune diseases, metabolic disorders, infectious diseases, and neurological conditions. In conclusion, LACC1 serves as a pivotal immune-metabolic hub, offering new insights and potential therapeutic targets for the study of related diseases.

## 1 Introduction

Laccases (alternatively referred to as poly-copper oxidoreductases) is a class of poly-copper oxidase widely distributed in fungi, plants and bacteria. Owing to its catalytic capacity to oxidize diverse phenolic and non-phenolic compounds, laccase has attracted considerable attention in the fields of lignin degradation, environmental pollution remediation, and industrial catalysis (1–3). A new class of laccase-domain proteins, Laccase-containing domain 1 (LACC1, previously called C13orf31 or FAMIN), has been discovered in mammals in recent years (4). LACC1 is named for its sequence homology to the laccase enzyme family (4), and may be related to fatty acid synthesis, purine nucleotide cycle, polyamine metabolism, and immune metabolism (4–8).

LACC1 is predominantly expressed in inflammatory macrophages (4, 9), and plays a critical role in autophagy, inflammatory responses, and bacterial clearance by integrating lipid metabolism, purine metabolism, and polyamine metabolism, thereby maintaining immune homeostasis (5, 7, 10). Furthermore, LACC1 has been shown to be closely associated with various systemic diseases, including immune disorders such as inflammatory bowel disease (IBD) and juvenile idiopathic arthritis (JIA) (6, 11), metabolic conditions such as non-alcoholic fatty liver disease(NAFLD) and atherosclerosis (AS) (12, 13), infectious diseases like leprosy (14), and neurological disorders such as cognitive impairment following stroke (15). Given LACC1’s pivotal regulatory function in disease onset and progression, this paper systematically elucidates its molecular mechanisms and disease correlations, thereby significantly advancing both basic research and clinical translation in relevant pathologies.

## 2 The structure of LACC1

Laccase is formed by the folding of three copper redoxin domains (T1, T2, and T3) (1), among which the T1 blue copper site is the main site for laccase to perform biological functions (1). LACC1 is a highly conserved 47-kD protein (6), mainly present on chromosomes 3, 6 (n=2), and 13 (16, 17). LACC1 contains a T1 blue copper site that is homologous to the T1 blue copper site found in laccases (4, 7, 10, 18), and the position of LACC1 variants are mostly concentrated in the T1 blue copper site (6). The human LACC1 gene lacks homology with other mammalian protein families (4). LACC1 is homologous to the functionally unknown domain 152 (DUF152) of the Pfam motif, which is similar to the bacterial proteins YlmD and YfiH (4, 7, 10, 18).

## 3 LACC1-mediated metabolism and function

More and more studies suggested that LACC1 presents a variety of enzymatic catalytic functions, including *de novo* lipogenesis (DNL) (6, 9), fatty acid oxidation (FAO) (9), purine nucleotide cycle (PNC) (7, 9), endoplasmic reticulum(ER)stress response (19), isocyanic acid synthesis, and polyamine metabolism (5, 8). These LACC1-mediated metabolic processes play an essential role in regulating functions such as inflammation, immune response, and autophagy (16, 19–22). Thus, how does LACC1 serve as a metabolic hut and exert its functions?

### 3.1 LACC1 and lipid metabolism

LACC1 participates in cellular metabolism by regulating lipid synthesis, decomposition and storage, and its function in macrophages has been relatively clearly studied. In terms of lipid synthesis, LACC1 forms a complex with fatty acid synthase (FASN) on the peroxisome and promotes metabolic flux through DNL. In terms of lipid decomposition, LACC1 drives high levels of FAO and glycolysis, thereby promoting ATP regeneration. Cell experiments confirmed that bone marrow-derived macrophages (BMDMs) deficient in LACC1 showed significantly reduced glycolysis, FAO and phosphocreatine (10). In addition to macrophages, LACC1 regulates similar lipid synthesis and decomposition processes in chondrocytes (23) and T cells (24), but no direct association has been found between it and lipid droplet synthesis in these two types of cells so far. In terms of lipid storage, lipid droplets are key structures for storing fatty acids within cells. A deficiency of LACC1 can lead to a significant reduction in lipid droplet synthesis in macrophages (25). In addition, LACC1-dependent DNL and FAO can regulate inflammasome activation, mitochondrial and NADPH oxidase-dependent reactive oxygen species (ROS) production, and the bactericidal activity of macrophages (10).

### 3.2 LACC1 and purine metabolism

LACC1 is a multifunctional purine nucleosidase that combines the activities of adenosine deaminase (ADA), purine nucleosidase phosphorylase (PNP), and S-methyl-5 ‘-thioadenosine phosphorylase (MTAP) (7). The enzymatic activity of LACC1 enables the formation of a purine nucleotide cycle (PNC) among adenosine, inosine monophosphate and adenosine succinic acid in macrophages. LACC1-dependent PNC regulates the cytoplasmic - mitochondrial REDOX balance, thereby preventing cytoplasmic acidification (7). The absence of ADA and PNP can lead to severe combined immune deficiency (26). Given that ADA and PNP are key enzymes in purine metabolism, LACC1-mediated purine metabolism may also be involved in immune regulation.

### 3.3 LACC1 and polyamine metabolism

Wei et al. demonstrated that LACC1 is crucial for polyamine metabolism in M1-type macrophages, primarily through its interaction with nitric oxide synthase (NOS2) and L-ornithine (L-Orn) in mediating polyamine immunity (5). The mechanism involves NOS2 converting L-arginine (L-Arg) into L-citrulline (L-Cit) and nitric oxide (NO), followed by LACC1 cleaving L-Cit to yield L-Orn and isocyanic acid (HNCO). The resulting L-Orn facilitates the synthesis of polyamines via the action of ornithine decarboxylase 1 (ODC1), ultimately leading to anti-inflammatory and antibacterial effects (5, 8, 27). Additionally, HNCO, generated by LACC1, can modulate inflammatory signals: it can aminoformylate NLRP3 at K593, disrupting the interaction between NLRP3 and NEK7 within the NLRP3 inflammasome, thereby limiting the activation of the NLRP3 inflammasome in LPS-induced macrophages (27).

### 3.4 LACC1 mediates metabolism-autophagy regulation

LACC1 enhances autophagy flux and promotes lysosomal degradation through interaction with autophagy inducers (receptor for activated C kinase1) RACK1 and AMPK (AMP-activated protein kinase), thereby maintaining the balance of cellular energy metabolism (5, 6, 28). Anne-Laure Mathieu et al. discovered that overexpression of LACC1 in Hela cells leads to an increase in the number of autophagosomes (25). Conversely, macrophages lacking LACC1 have a reduced ability to take up apoptotic bodies (5, 6, 25, 29). In addition, autophagy-associated protein 5 (ATG5) is a key component of autophagy (30). The reduction of autophagosomes in LACC1 deficiency and ATG5 deficiency is consistent (25). Accordingly, LACC1 plays an important role in autophagy and energy balance of cells.

### 3.5 LACC1 mediates metabolism-immune regulation

LACC1 serves as a pivotal regulatory factor in immune metabolism (31), crucial for preserving immune balance, orchestrating infection defense through the integration of metabolic pathways and immune reactions. Furthermore, dysfunctions in LACC1 functionality have been linked to various immune disorders (32).

#### 3.5.1 LACC1 and innate immunity

During innate immunity, LACC1 primarily regulates the metabolic state and functions of effector cells, including macrophages and neutrophils, thereby establishing the first line of defense against pathogen clearance and facilitating the transition to adaptive immunity.

LACC1 is prominently expressed in inflammatory macrophages and serves as a crucial regulatory factor for antibacterial defense (6, 8, 28, 33). Cellular studies have demonstrated that LACC1 enhances the antibacterial capacity of macrophages by modulating the NOD2-ER stress signaling pathway and the L-Orn-polyamine immune metabolic signaling axis (5, 16). Additionally, animal studies revealed that the abundance of intestinal flora in LACC1 knockout (KO) mice significantly increased, leading to an exacerbation of the microbial load (5, 11). Thus, LACC1 may play a role in innate immunity by augmenting the antibacterial activity of macrophages.

The expression of LACC1 enhances the phagocytic function of neutrophils (33). Research indicates that ADP-ribosylation factor 6 (Arf6) deficiency results in the down-regulation of LACC1, leading to significant decreases in glycolysis, ROS production, and phagocytosis in neutrophils (33). This finding aligns with the metabolic regulatory role of LACC in promoting glycolysis and ROS production. Consequently, LACC1 may influence neutrophil function via metabolic regulation, thereby playing a role in innate immunity.

#### 3.5.2 LACC1 and adaptive immunity

Although LACC1 is lowly expressed or almost not expressed in lymphocytes (4, 34), its regulatory role in adaptive immunity cannot be overlooked, mainly by influencing the functions of dendritic cells, T cells and B cells.

LACC1 is a crucial molecule in dendritic cells (DCs) that regulates T cell differentiation. When LACC1-deficient bone marrow-derived dendritic cells (BMDCs) are co-cultured with T cells, the levels of Th1/Th17 cytokines (IFN-γ and IL-17) decrease, while Th2 cytokines (IL-4) increase. This pattern is consistent with observations made when DCs derived from mesenteric lymph nodes (MLN) lacking LACC1 are co-cultured with T cells. Furthermore, the induction of lipid A leads to a reduction in the expression of surface co-stimulatory molecules (CD40, CD80, CD86, ICAM-1) in LACC1-deficient BMDCs. Supplementation with these molecules can restore the normal cytokine secretion pattern of T cells (16). Thus, LACC1 may play a role in adaptive immunity by modulating the expression of co-stimulatory molecules in DCs and influencing T cell polarization.

LACC1 is expressed in T cells and plays a crucial role in adaptive immunity. Its regulation of T cells exhibits disease-specific variations. In the inflammatory bowel disease model, LACC1 KO mice developed more severe T-cell metastatic colitis, characterized by low levels of Th1/Th17 cytokines. This deficiency led to impaired T-cell immune responses and an increased burden on the intestinal microbiota (16). Conversely, in the arthritis model involving LACC1 KO mice, collagen II induced Th17 cell differentiation and a significant rise in IL-17, which resulted in impaired immune tolerance and facilitated the onset of autoimmunity (4, 6). This seemingly contradictory phenomenon may be attributed to differences in the cytokine profiles present in the disease microenvironment. Furthermore, LACC1 possesses extensive regulatory functions in T-cell metabolism (13, 24). The fatty acid and glycolytic reserves in CD4+ T cells with LACC1 knockdown are diminished (24). Thus, LACC1 may influence adaptive immunity by mediating T cell differentiation and metabolic processes.

LACC1 is linked to various autoimmune disorders (31). Notably, a strong positive relationship exists between LACC1 and autoantibodies in individuals who have recovered from leprosy (31). This association implies that LACC1 might modulate B cell activation or antibody production in the immune response. However, the precise role of LACC1 in B lymphocytes is not yet fully understood, necessitating further investigation.

## 4 LACC1 and signaling pathway

The expression of LACC1 is subject to various influences. Research indicates that LACC1 expression is elevated in pathological states like surgery under anesthesia and stroke (15, 35). Furthermore, microorganisms and their constituents, including Mycobacterium leprae (14), lipopolysaccharide (LPS) (5, 11, 34), muramyl dipeptide (MDP) (36), and polyinosinic: polycytidylic acid (poly-I:C) (5, 11), can activate PRRs, prompting LACC1 expression in macrophages (36). Moreover, multiple upstream regulatory elements like TGF-β1 (20), Arf6 (33), INF-γ (34), and GM-CSF (6) directly control LACC1 expression. Nevertheless, the precise mechanisms through which these upstream regulators interact with LACC1 remain unclear.

LACC1 is involved in multiple signaling pathways and plays a role in processes such as inflammatory regulation, autophagy, and microbial clearance.

### 4.1 M-CSF/AKT/mTOR/LACC1 signaling pathway

The M-CSF-AKT-mTOR signaling pathway is mainly related to autophagy (6). Upon stimulation by macrophage colony-stimulating factor (M-CSF), M-CSF binds to the colony-stimulating factor 1 receptor (CSF1R), which promotes Phosphatidylinositol 3-Kinase (PI3K)- Protein Kinase B (AKT) signaling and subsequently activates mechanistic target of rapamycin (mTOR). mTOR significantly enhances the expression of LACC1, thereby facilitating autophagy (6). Inhibitors targeting the AKT-mTOR pathway have been shown to reduce LACC1 expression in macrophages (6). Furthermore, studies indicate that LACC1 deficiency can result in aberrantly elevated mTOR activity, suggesting that LACC1 may play a role in regulating mTOR signaling through a negative feedback mechanism and could serve as a potential regulatory factor in the activation of the autophagy cycle (6). Consequently, the M-CSF/AKT/mTOR pathway is the primary signaling route for LACC1 expression; however, the precise transcriptional mechanism by which mTOR regulates LACC1 remains unclear.

### 4.2 PPARα/RXR/PPRE/LACC1 signaling pathway

The PPARα/RXR/PPRE/LACC1 signaling pathway is mainly involved in inflammation. Peroxisome proliferator-activated receptor alpha (PPARα) forms a heterodimer with the retinoid X receptor (RXR), which subsequently binds to the PPAR response element (PPRE) to activate downstream gene transcription and then inhibits the expression of LACC1, reducing inflammatory injury and pyroptosis (22). Furthermore, since both PPARα and LACC1 promote FAO, it has been suggested that the mechanism of PPARα-mediated downregulation of LACC1 may be related to a potential feedback loop controlling the FAO rate (22). However, the precise mechanism by which PPARα regulates LACC1 remains unclear.

### 4.3 miR-211-5p/KDM2B/LACC1 signaling pathway

The miR-211-5p/KDM2B/LACC1 signaling pathway is mainly involved in inflammation. miR-211-5p targets and inhibits the expression of histone lysine demethylase (KDM2B), which promotes the methylation of H3K4me3 in the LACC1 promoter region. This process facilitates LACC1 transcription and ultimately suppresses excessive inflammatory responses (37). Animal studies have demonstrated that the miR-211-5p/KDM2B/LACC1 axis can mitigate tissue damage induced by inflammation, suggesting it as a potential target for the intervention of inflammatory diseases (37).

### 4.4 LACC1/AMPK/NLRP3 signaling pathway

The LACC1/AMPK signaling pathway is primarily associated with inflammation. Upregulation of LACC1 expression significantly inhibits AMPK phosphorylation, alleviates AMPK’s suppression of the NOD-like receptor pyrin domain-containing 3 (NLRP3) inflammasome, and exacerbates the inflammatory response (15). However, the regulatory relationship between LACC1 and AMPK remains contentious across different pathways. In a macrophage autophagy model, starvation did not induce a significant difference in AMPK phosphorylation levels compared to LACC1 knockdown macrophages, indicating that LACC1 may function downstream of AMPK within the autophagy pathway. Further investigation is required to elucidate the mechanisms underlying this regulatory discrepancy (6).

### 4.5 LACC1/NF-κB signaling pathway

The LACC1/NF-κB signaling pathway is implicated in pyroptosis, inflammation, and bacterial clearance. Under NOD2 stimulation, LACC1 associates with the NOD2 signaling complex, which includes NOD2, RIP2, IRAK1, TRAF6, p-ERK, p-p38, and p-IκBα, thereby activating the nuclear factor kappa-B (NF-κB) signaling pathway (36). Additionally, LACC1 interacts with the subunit A of succinate dehydrogenase, enhancing ROS production induced by PRRs and indirectly activating NF-κB (36). Experimental evidence indicates that LACC1 expression in macrophages significantly increases under LPS stimulation, counteracting the inhibitory effect of lupin alcohol on NF-κB and promoting macrophage polarization and pyroptosis (22). Furthermore, LACC1 deficiency can impair NF-κB-dependent production of ROS, RNS, and autophagy-mediated bacterial clearance (16, 36). However, it remains perplexing that some studies have reported that LACC1 deficiency in human macrophages does not influence inflammasome activation or NF-κB signaling during inflammatory responses (6). The underlying mechanism of this discrepancy has yet to be fully elucidated.

### 4.6 LACC1/MAPK signaling pathway

The LACC1/MAPK signaling pathway is mainly involved in inflammation and bacterial clearance. With PRRs stimulation, LACC1 is up-regulated and assembles into a complex with the downstream proteins of PRR, such as TRAF6 and IRAK1, which activates the downstream mitogen-activated protein kinase (MAPK) pathway of the complex, thereby expanding the inflammatory response and participating in bacterial clearance (16, 19). On the contrary, LACC1 expression is down-regulated in patients with LACC1 mutations, resulting in reduced MAPK pathway activation, decreased secretion of inflammatory cytokine, and lower bacterial clearance (19, 36). To date, no direct interaction between LACC1 and MAPK has been identified.

### 4.7 LACC1/ROS/NOD2/PIPK2 signaling pathway

The LACC1/ROS/NOD2/PIPK2 pathway is primarily linked to inflammatory activation and microbial clearance. LACC1 facilitates NOD2-induced ROS generation by interacting with the A subunit of succinate dehydrogenase (SDH) (36). This ROS production enhances the formation of the NOD2-RIPK2 complex, activates the NOD2 signaling pathway, and subsequently promotes cytokine secretion and bacterial clearance (16, 35, 36).

### 4.8 LACC1/JAK-STAT signaling pathway

The LACC1/JAK-STAT signaling pathway is primarily associated with inflammation. The Janus kinase-signal transducer and activator of transcription (JAK-STAT) signaling pathway facilitates the transduction of inflammatory signals (38). Notably, overexpression of LACC1 in the extracellular vesicles of macrophages significantly decreases the phosphorylation levels of JAK2/STAT3 and the enrichment of related genes, indicating that LACC1 inhibits the JAK-STAT inflammatory pathway (23). Nevertheless, the precise mechanism underlying the interaction between LACC1 and JAK/STAT remains unclear.

### 4.9 LACC1/CCL5 signaling pathway

LACC1/CCL5 signaling pathway is mainly associated with inflammation. Research indicates that loss-of-function variants of LACC1, such as P. Glu348TER, result in the upregulation of chemokine (C-C motif) ligand 5 (CCL5), thereby enhancing the secretion of inflammatory factors (39). Currently, this relationship remains a preliminary correlation. The precise mechanism by which LACC1 regulates CCL5 requires further experimental validation.

## 5 LACC1 and diseases

### 5.1 LACC1 and immune diseases

The LACC1 gene has been identified through genome-wide association studies (GWAS) as a prevalent risk factor for several autoimmune diseases, including JIA (40–44), rheumatoid arthritis (RA) (45), Behcet’s disease (BD) (4, 9, 46–48), and psoriasis (4, 11). Currently, research on LACC1 predominantly centers on IBD and JIA, while investigations into its mechanisms in other diseases remain in the early stages.

#### 5.1.1 Inflammatory bowel disease

IBD is a chronic inflammatory disorder of unknown etiology, primarily characterized by Crohn’s disease (CD) and ulcerative colitis (UC) (49). Over recent decades, the global incidence of IBD has steadily increased, significantly impacting patients’ quality of life (50, 51). GWAS and meta-analyses have identified multiple locus variations in the LACC1 gene, including rs3764147 and rs1467526, which are significantly associated with susceptibility to IBD, particularly among patients with CD (21, 34, 43, 52, 53).

LACC1 serves as a multifunctional hub for maintaining intestinal homeostasis. On the one hand, LACC1 promotes polyamine synthesis via the L-Orn -polyamine axis and enhances the intestinal bacterial clearance rate (4, 5). On the other hand, LACC1-dependent ER stress can amplify PRRs-induced NF-κB and MAPK signaling in macrophages, activate the ROS pathway, the RNS pathway, and the autophagy pathway, thereby facilitating the elimination of invading pathogens (19).

When LACC1 is dysfunctional, the balance of intestinal flora is disrupted, resulting in dysbiosis and an excessive release of inflammatory factors, such as TNF-α and IL-6 (11, 16, 19). Furthermore, LACC1 deficiency may weaken the function of medullary Th1/Th17 cells, thereby reducing the body’s immune surveillance of the intestinal flora (16). More importantly, there is functional compensation within the ER stress and autophagy pathways associated with LACC1. The simultaneous impairment of both pathways can directly lead to severe Crohn’s disease-like ileitis (54).

In conclusion, LACC1 plays a crucial role in preventing the occurrence and development of IBD by coordinating immune metabolism.

#### 5.1.2 Juvenile idiopathic arthritis

JIA is a chronic and heterogeneous form of arthritis with an unknown etiology that manifests during childhood (25, 55, 56). Only 20-25% of affected individuals attain complete remission, while the majority confront the possibility of lifelong recurrence, significantly impairing their quality of life (56). The relationship between LACC1 and JIA has been validated through several GWAS (17, 39–44).

Clinical data indicate that patients with JIA exhibit LACC1 mutations, which are associated with reduced expression levels of this gene (57). Recent investigations have revealed that LACC1 deficiency leads to impaired autophagy, a condition closely linked to the onset of JIA (6, 58). Specifically, the autophagy process dependent on LACC1 facilitates the formation of lipid droplets, which chelate excess fatty acids in the joint area. This mechanism limits the accumulation of cytotoxic lipids and mitigates joint damage caused by inflammation and oxidative stress. Additionally, the stored fatty acids serve as a source of energy for cellular metabolism through mitochondrial respiration (4, 6, 25).

LACC1 mitigates inflammatory damage in juvenile idiopathic arthritis (JIA). Specifically, LACC1 reduces the inflammatory response associated with JIA through autophagy (4, 6, 25). Additionally, LACC1 diminishes the aggregation of inflammatory cells within the synovium of JIA patients and inhibits the degradation of the extracellular matrix in synovial fibroblasts by targeting the CCL5/CCR5 axis, thereby lessening joint damage (39). Conversely, some researchers argue that LACC1 may act as a trigger for inflammation in JIA. Notably, elevated levels of complement C5 have been detected in patients with LACC1 mutations. Following the cleavage of C5 into C5a, the resultant C5A-des Arg predominantly binds to the anti-inflammatory receptor C5aR2, which inhibits pro-inflammatory signaling mediated by C5AR1 through the ERK1/2 pathway (39). Current research predominantly supports the anti-inflammatory role of LACC1 in JIA. The observed contradiction may stem from variations in the cellular microenvironment and crosstalk among signaling pathways, warranting further investigation.

#### 5.1.3 Rheumatoid arthritis

RA is an autoimmune disease marked by synovitis and progressive joint destruction (59). In a clinical cohort study, Zhou Yang et al. observed that elevated expression levels of LACC1 in patients were positively correlated with the efficacy of the drug upadacitinib (UPA). This finding suggests that LACC1 may serve as a potential biomarker for treatment response in RA (45).

#### 5.1.4 Behçet’s disease

BD is a chronic systemic vasculitis primarily characterized by systemic inflammation mediated by innate immunity (60, 61). In a mouse model with a knockout of the LACC1 gene, the production of IL-1β was found to decrease in response to LPS stimulation, aligning with the established role of IL-1β in the pathogenesis of BD (46, 48). Although GWAS have identified LACC1 as a risk gene for BD (4, 9, 46–48, 62), the specific protein function and mechanism of action of LACC1 in the disease remain to be elucidated.

#### 5.1.5 Psoriasis

Psoriasis is a chronic inflammatory skin disease mediated by the immune system, characterized by abnormal proliferation and differentiation of epidermal cells (63). Mutations in the LACC1 gene are linked to an increased susceptibility to psoriasis (4, 11). In disease models, the absence of LACC1 exacerbates psoriasis-like symptoms (4, 5), suggesting that this gene plays a protective role in disease regulation and offering a novel avenue for therapeutic research.

### 5.2 LACC1 and metabolic diseases

#### 5.2.1 Non-alcoholic fatty liver disease

NAFLD is the primary cause of chronic liver diseases globally. Its development is closely linked to disorders in lipid metabolism, oxidative stress, and inflammatory responses within the liver (12, 64). Research indicates that LACC1 is down-regulated in both NAFLD patients and mouse models, positioning it as a potential biomarker for disease prediction (12). Subsequent mechanistic studies have demonstrated that LACC1 enhances lipid metabolism by facilitating the FAO process (10). Consequently, the lipid metabolism disorders associated with LACC1 are intricately connected to the pathological progression of NAFLD.

#### 5.2.2 Atherosclerosis

AS is a chronic inflammatory condition mediated by the immune system and characterized by disorders in lipid metabolism (65). Research involving animal models has demonstrated that LACC1 can impede the progression of AS by enhancing polyamine immune metabolism in inflammatory macrophages, thereby suppressing lipid accumulation and inflammatory responses (13). Consequently, LACC1 may serve as a promising therapeutic target for AS.

### 5.3 LACC1 and infectious diseases

#### 5.3.1 Leprosy

Leprosy is a chronic infectious disease caused by infection with Mycobacterium leprae, primarily affecting the skin and peripheral nervous system (66). GWAS have identified LACC1 as a core susceptibility gene for leprosy across various populations (14, 21, 67–71). Notably, the mRNA expression level of LACC1 is upregulated in the lesion tissues of patients and in cells stimulated by Mycobacterium leprae (14), which contradicts the typical expectation of decreased expression resulting from gene mutations. The underlying mechanism remains to be elucidated.

Research indicates that LACC1 may facilitate bacterial immune evasion through metabolic reprogramming. Specifically, upon activation by Mycobacterium leprosy, LACC1 promotes glycolysis and lipid synthesis in host cells, thereby aiding bacteria in evading clearance from the lysosomal/proteasome system by inducing mitochondrial autophagy and disrupting key molecules involved in xenophagocytosis (72). Furthermore, clinical observations reveal that autoantibodies persistently detectable in the bodies of cured leprosy patients exhibit a positive correlation with LACC1 expression (31). This finding suggests that LACC1 may function as an immunogenic molecule, contributing to the sustained activation of the autoimmune response following infection. Additionally, the expression of LACC1 in patient tissues correlates positively with the production of ROS and the activation level of the NLRP3 inflammasome (72). This correlation implies that LACC1 may enhance ROS accumulation by modulating lipid metabolism, thereby activating the NF-κB/NLRP3 pathway and exacerbating tissue inflammation and nerve damage.

### 5.4 LACC1 and neuropsychiatric disorders

#### 5.4.1 Sepsis-induced neuroinflammation

Sepsis-induced neuroinflammation represents an inflammatory response of the nervous system initiated by infectious stimuli during sepsis, potentially resulting in neuronal damage and neurological dysfunction (73). Experimental studies have shown that NOD2 stimulation can enhance the expression of LACC1 in microglia, exacerbate endoplasmic reticulum stress, and consequently promote the neuroinflammatory process (73).

#### 5.4.2 Cognitive dysfunction

LACC1-related cognitive dysfunction primarily encompasses anesthesia-induced cognitive impairment and post-stroke cognitive impairment. Neuroinflammation and oxidative stress represent common underlying mechanisms (15, 35, 74).

In models of anesthesia-induced cognitive impairment, the expression of LACC1 in mouse brain tissue is elevated. This increase activates the LACC1/mROS signaling pathway, which induces the aggregation of NOD2 and the formation of NOD2-RIP2 complexes, ultimately leading to mitochondrial dysfunction in neurons and exacerbating cognitive deficits (35).

Similarly, in cases of stroke-related cognitive impairment, LACC1 expression is also upregulated in cerebral ischemic tissues. This upregulation promotes inflammation and oxidative stress via the AMPK/NLRP3 signaling pathway, mediates the death of neurons and glial cells, and further aggravates cognitive impairment (15).

These findings suggest that LACC1 may exert neurotoxic effects in cognitive impairment, and the associated signaling pathways could serve as potential therapeutic targets for related disorders.

### 5.5 LACC1 and fibrotic diseases

The primary pathological characteristics shared by autoimmune myocarditis, Frozen shoulder (FS), and Temporomandibular joint osteoarthritis (TMJOA) include chronic inflammation that leads to tissue damage, along with dysregulated repair mechanisms such as fibrosis. LACC1 is involved in the disease process by modulating inflammation and inhibiting fibrosis-related damage, among other pathways.

#### 5.5.1 Autoimmune myocarditis

Autoimmune myocarditis is primarily defined by the infiltration of inflammatory cells into the myocardium, which leads to myocardial cell necrosis and fibrosis (75, 76). Research indicates that the down-regulation of LACC1 expression, mediated by PPARα, can inhibit the NF-κB/NLRP3 inflammatory signaling pathway. This inhibition subsequently reduces macrophage polarization to the M1 phenotype and pyroptosis, ultimately mitigating the degradation and fibrosis of myocardial tissue (22).

#### 5.5.2 Frozen shoulder

FS is characterized by inflammation surrounding the joint, which leads to progressive pain and restricted shoulder movement (37, 77). Prolonged inflammation results in fibrosis of the synovial sac, ultimately causing irreversible damage (77, 78). Experimental studies in animals have demonstrated that miR-211-5p, present in extracellular vesicles derived from bone marrow mesenchymal stem cells, can mitigate FS in rats by modulating the KDM2B/LACC1 axis. The activation of LACC1 inhibits the overexpression of inflammatory factors(TGF-β) and tissue remodeling factors(MMP1 and MMP3). Additionally, it corrects the abnormal expression of nerve repair factors(GAP43 and PGP9.5)thereby enhancing joint function and facilitating tissue repair (37).

#### 5.5.3 Temporomandibular joint osteoarthritis

TMJOA is characterized by cartilage degeneration, chronic pain, and joint dysfunction. Research indicates that engineered extracellular vesicles (OE-EV) overexpressing LACC1 can significantly inhibit IL-1β-induced inflammatory responses, enhance mitochondrial function, decrease lactic acid production and superoxide levels, mitigate cartilage matrix degradation, and remodel subchondral bone (23).

### 5.6 LACC1 and tumors

#### 5.6.1 Colorectal cancer

Colorectal cancer (CRC) is a prevalent malignant tumor globally. A significant challenge in its treatment arises from the propensity of cancer cells to undergo distant metastasis, which adversely affects patient prognosis (20, 79). Recent studies indicate that the TGF-β signaling pathway may influence the purine metabolism of CRC cells by modulating the expression of LACC1. This regulation leads to the production of substantial amounts of inosine, which promotes epithelial-mesenchymal transition (EMT), enhances cell migration, and accelerates cancer progression (20). Nevertheless, the precise mechanism by which LACC1 operates in colorectal cancer remains unclear, necessitating further verification through *in vivo* experiments.

## 6 Conclusions

**Figure 1:**
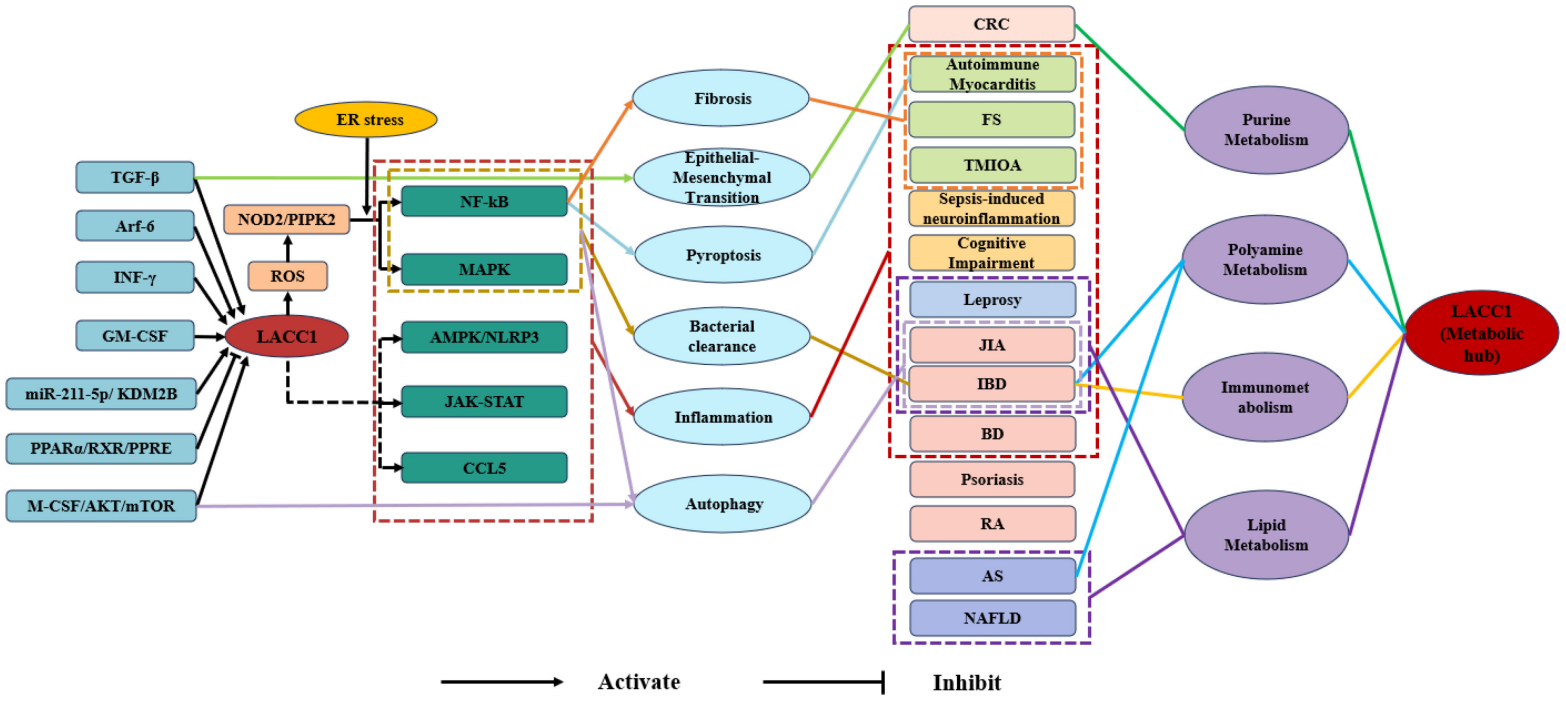
Mechanistic diagram of LACC1-mediated signaling pathways, metabolic regulation, and disease correlations. TGF-β, Transforming Growth Factor-β; Arf-6, ADP-Ribosylation Factor 6; INF-γ, Interferon-γ; GM-CSF, Granulocyte-Macrophage Colony-Stimulating Factor; miR-211-5p, microRNA-211-5p;KDM2B, Lysine Demethylase 2B; PPARα, Peroxisome Proliferator-Activated Receptor α; RXR, Retinoid X Receptor; PPRE, Peroxisome Proliferator Response Element; M-CSF, Macrophage Colony-Stimulating Factor; AKT, Protein Kinase B; mTOR, Mammalian Target of Rapamycin; NOD2, Nucleotide-Binding Oligomerization Domain Containing 2; PIPK2, Phosphatidylinositol Phosphate Kinase 2; ROS, Reactive Oxygen Species; LACC1, Laccase Domain Containing 1; NF-κB, Nuclear Factor Kappa-B; MAPK, Mitogen-Activated Protein Kinase; AMPK, AMP-Activated Protein Kinase;NLRP3, NOD-Like Receptor Family Pyrin Domain Containing 3; JAK-STAT, Janus Kinase-Signal Transducer and Activator of Transcription; CCL5, Chemokine (C-C Motif) Ligand 5; CRC, Colorectal Cancer; FS, Frozen Shoulder; TMJOA, Temporomandibular Joint Osteoarthritis JIA, Juvenile Idiopathic Arthritis; IBD, Inflammatory Bowel Disease; BD, Behcet’s Disease; RA, Rheumatoid Arthritis; AS, Atherosclerosis; NAFLD, Non-Alcoholic Fatty Liver Disease.

Current research has confirmed that LACC1 possesses a T1 blue copper site homologous to laccase, with a highly conserved structure. LACC1 exhibits multiple enzymatic functions and regulates lipid synthesis, decomposition, and storage processes within cells, such as macrophages, in lipid metabolism. In purine metabolism, it functions as a multifunctional purine nucleosidase, participating in the purine nucleotide cycle. Regarding polyamine metabolism, LACC1 links NOS2 to polyamine metabolism, thereby exerting anti-inflammatory and antibacterial effects. At the signaling pathway level, LACC1 is implicated in various signaling pathways and plays a crucial role in processes such as autophagy, inflammatory regulation, and bacterial clearance. In disease research, LACC1 is closely associated with the pathogenic mechanisms of immune diseases (such as IBD and JIA), metabolic diseases (such as NAFLD and AS), infectious diseases (such as leprosy), neuropsychiatric disorders (such as sepsis-induced neuroinflammation and cognitive dysfunction), and fibrotic and degenerative diseases (such as autoimmune myocarditis and periarthritis of the shoulder). Some studies have elucidated its specific mechanisms of action in disease occurrence and progression. For instance, LACC1 is involved in the pathogenesis of IBD through mechanisms such as the L-ornithine-polyamine axis. The core functional mechanisms of LACC1 are illustrated in **Figure 1**.

Although the association between LACC1 and various diseases has been well established, the specific mechanisms underlying its action in different conditions remain incompletely understood. For example, in Behcet’s disease and psoriasis, the functional role and mechanism of action of LACC1 require further elucidation. Regarding signaling pathways, the regulatory relationship between LACC1 and AMPK is contentious across different pathways. Additionally, the role of LACC1 in regulating NF-κB within the LACC1/NF-κB signaling pathway is also debated. The mechanisms by which M-CSF-AKT-mTOR and PPARα/RXR/PPRE regulate LACC1 are not yet clarified. Furthermore, the LACC1/JAK-STAT and LACC1/CCL5 signaling pathways are currently only tentatively associated, and their precise mechanisms necessitate experimental validation. Furthermore, the functions of LACC1 in cell types beyond those investigated, such as B cells and chondrocytes, as well as under various physiological and pathological conditions, remain speculative, and its associated roles have yet to be clarified.

Future research should prioritize the biochemical characterization of LACC1 activity and thoroughly investigate the specific molecular mechanisms through which it performs enzymatic functions under diverse physiological and pathological conditions. By utilizing high-throughput screening and other advanced technologies, researchers aim to identify small molecule compounds capable of modulating LACC1 activity to establish the groundwork for developing targeted drugs centered on LACC1. Clinical studies have validated the significant involvement of LACC1 in various diseases, highlighting its potential as a predictor of NAFLD and a determinant of UPA efficacy. Subsequent investigations should aim to validate the feasibility of targeting LACC1 as a treatment strategy and a biomarker in extensive clinical cohorts, elucidate its clinical relevance in disease diagnosis, prognosis evaluation, and treatment response monitoring, and advance the spectrum of LACC1 research from fundamental science to clinical implementation.

## Glossary

LACC1: Laccase-containing domain 1
DUF152: unknown domain 152
DNL: *de novo* lipogenesis
FAO: fatty acid oxidation
PNC: purine nucleotide cycle
ER: endoplasmic reticulum
ROS: reactive oxygen species
mROS: mitochondrial reactive oxygen species
ODC1: ornithine decarboxylase 1
BMDMs: bone marrow-derived macrophages
DC: dendritic cells
ATG5: autophagy-associated protein 5
M-CSF: macrophage colony stimulating factor
CSF1R: colony stimulating factor 1 receptor
PI3K: PI-3 kinase
PIP2: phosphatidylinositol-4,5-bisphosphate
PIP3: phosphatidylinositol-3,4,5- trisphosphate
AKT: protein kinase B
mTOR: mammalian target of rapamycin
PPARα: peroxisome proliferator activated receptor α
RXR: retinol X receptor
PPRE: peroxisome proliferative reaction element
KDM2B: Lysine-specific demethylase 2B
AMPK: AMP-dependent protein kinase
Arf-6: ADP-ribosylation factor 6
TGF-β: transforming growth factor-β
NF-κB: nuclear factor κB
MAPK: mitogen-activated protein kinase
mROS: mitochondrial reactive oxygen species
RNS: active nitrogen
CCL5: chemokine ligand 5
EVs: extracellular vesicles
BMSCs: bone marrow mesenchymal stem cells
SDH: succinate dehydrogenase
NOS2: nitric oxide synthase 2
LPS: lipopolysaccharide
MDP: muramyl dipeptide
poly-I: C: polyinosinic- polycytidylic acid
EMT: epithelial-mesenchymal transition
Arf6: ADP ribosylation factor 6
IBD: Inflammatory bowel disease
CD: Crohn’s disease
UC: ulcerative colitis
NAFLD: Non-alcoholic fatty liver disease
JIA: Juvenile Idiopathic Arthritis
C5: Complement 5
BD: Behcet disease
FS: Frozen shoulder.
